# Pristimerin Causes G1 Arrest, Induces Apoptosis, and Enhances the Chemosensitivity to Gemcitabine in Pancreatic Cancer Cells

**DOI:** 10.1371/journal.pone.0043826

**Published:** 2012-08-28

**Authors:** Yongwei Wang, Yinan Zhou, Haoxin Zhou, Guang Jia, Ji Liu, Bing Han, Zhuoxin Cheng, Hongchi Jiang, Shangha Pan, Bei Sun

**Affiliations:** Department of Pancreatic and Biliary Surgery, The First Affiliated Hospital of Harbin Medical University, Harbin, People’s Republic of China; University of Kansas Medical Center, United States of America

## Abstract

Despite rapid advances in chemotherapy and surgical resection strategies, pancreatic cancer remains the fourth leading cause of cancer related deaths in the United States with a 5-year survival rate of less than 5%. Therefore, novel therapeutic agents for the prevention and treatment of pancreatic cancer are urgently needed. The aim of this study was to investigate the effect of pristimerin, a quinonemethide triterpenoid compound isolated from Celastraceae and Hippocrateaceae, on inhibition of cell proliferation and induction of apoptosis in three pancreatic cancer cells, BxPC-3, PANC-1 and AsPC-1, in both monotherapy and in combination with gemcitabine. Treatment with pristimerin decreased the cell proliferation of all three pancreatic cancer cells in a dose- and time-dependent manner. Treatment of pancreatic cancer cells with pristimerin also resulted in G1-phase arrest which was strongly associated with a marked decrease in the level of cyclins (D1 and E) and cyclin-dependent kinases (cdk2, cdk4 and cdk6 ) with concomitant induction of WAF1/p21 and KIP1/p27. Pristimerin treatment also resulted in apoptotic cell death, cleavage of caspase-3, modulation in the expressions of Bcl-2 family proteins, inhibition of the translocation and DNA-binding activity of NF-κB. In addition, pristimerin potentiated the growth inhibition and apoptosis inducing effects of gemcitabine in all three pancreatic cancer cells, at least in part, by inhibiting constitutive as well as gemcitabine-induced activation of NF-κB in both its DNA-binding activity and transcriptional activity. Taken together, these data provide the first evidence that pristimerin has strong potential for development as a novel agent against pancreatic cancer.

## Introduction

Pancreatic cancer is one of the most aggressive and lethal human cancers, which has steadily increased in incidence over the past decades, and is currently the fourth leading cause of cancer death in the United States. The collective 1-year survival from the time of diagnosis at any stage is only 26%, while a long-term overall survival (OS) falls down to <5% [1]. In 2010, it was estimated 43,140 Americans were diagnosed with pancreatic cancer and 36,800 died of the disease [2]. The main reason for such poor prognosis of pancreatic cancer is mostly due to the lack of early diagnosis, the highly aggressive biological behavior of the tumor and the poor response to most therapies including chemotherapy, radiotherapy, and immunotherapy [3]. Currently, Gemcitabine (2′-deoxy-2′,2′-difluorocytidine) is known to be the mainstay in the treatment for advanced and metastatic pancreatic cancer. However, gemcitabine treatment results in a response rate of less than 20% and is associated with multiple adverse events and chemoresistance [4], [5]. Therefore, developing alternative chemopreventive and chemotherapeutic strategies are urgently needed for the treatment of this deadly disease.

Nuclear factor-κB (NF-κB),which plays a critical regulatory role in the expression of genes involved in inflammation, cell proliferation, invasion, angiogenesis, metastasis, suppression of apoptosis, is constitutively activated in a variety of cancer cells including pancreatic cancer cells [6], [7], [8]. In addition, Multiple lines of evidence suggest that NF-κB plays a pivotal role in the growth and chemoresistance of pancreatic cancer. First, NF-κB is constitutively activated in pancreatic cancer cells, but not in normal pancreatic tissues or nontumorigenic cell lines [7], [9]. Second, NF-κB could promote pancreatic cancer growth due to its ability to inhibit cell apoptosis, induce mitogenic gene products (c-Myc and cyclin D1), increase expression of proangiogenic factor including vascular endothelial growth factor(VEGF), and promote the migration and invasion of pancreatic cancer cells [10], [11], [12], [13], [14]. Finally, NF-κB plays a pivotal role in mediating chemoresistance in pancreatic cancer [15]. Together, this evidence implicates the role of NF-κB in pancreatic cancer and suggests agents that can block NF-κB activation have potential to combat the growth of pancreatic cancer.

**Figure 1:**
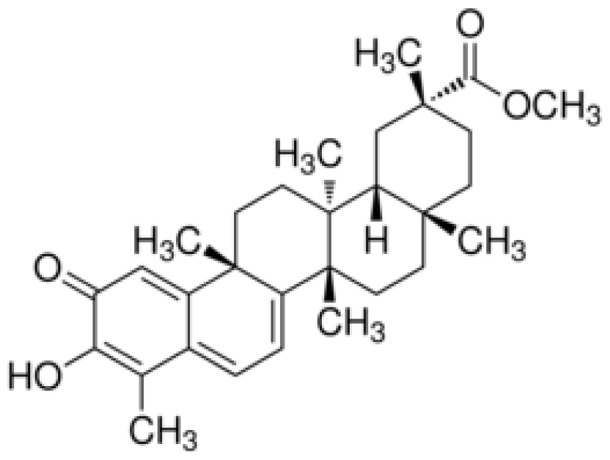
Chemical structure of pristimerin.

Pristimerin (Fig. 1), a naturally occurring quinonemethide triterpenoid compound isolated from Celastraceae and Hippocrateaceae, has recently attracted considerable interest due to its potential chemopreventive or chemotherapeutic properties. It has anti-inflammatory, antioxidant, antimalarial, and insecticidal activities and has been shown to possess growth inhibitory effect on a series of human cancer cell lines such as breast and lung cancer, prostate cancer, cervical cancer and multiple myeloma tumors [16], [17], [18], [19], [20]. However, the potential efficacy of pristimerin against pancreatic cancer remains unknown.

In the present study, we used three human pancreatic cancer cell lines, BxPC-3, PANC-1 and AsPC-1, to evaluate the potential of pristimerin as an effective chemopreventive and chemotherapeutic agent against pancreatic cancer. We demonstrate that pristimerin strongly suppresses the growth of all three pancreatic cancer cell lines by inducing G1-phase cell cycle arrest and apoptosis, and sensitizes them to gemcitabine-induced cell death. Furthermore, Our results demonstrate that the effects and molecular mechanisms of action of pristimerin in pancreatic cancer cells are associated with inhibition of NF-κB activation.

## Results

### Pristimerin Induces Cell Growth Inhibition in Pancratic Cancer Cells

**Figure 2:**
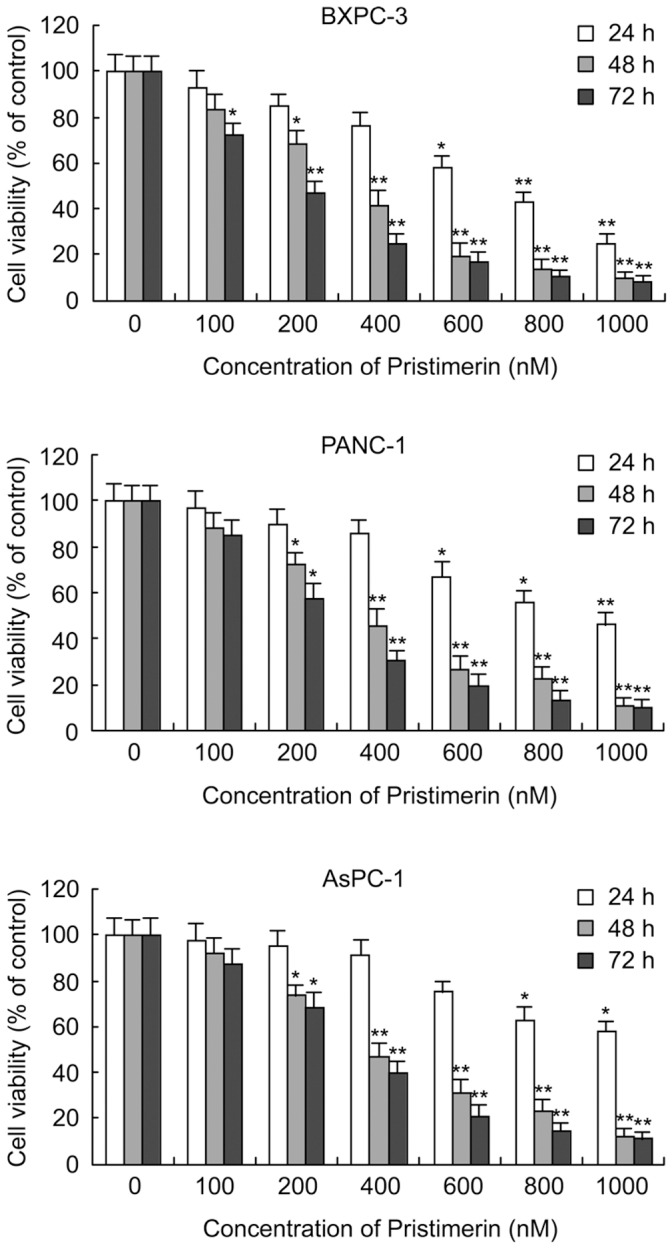
Effect of pristimerin on cell growth. BxPC-3, PANC-1 and AsPC-1 cells were grown in the absence or presence of increasing concentration of pristimerin for 24 h, 48 h and 72 h and then the viability of cells was measured by CCK-8 assay as described in Materials and Methods. Data shown are representative of at least three independent experiments. *P<0.05, compared with control. **P<0.01, compared with control.

To determine the effect of pristimerin on cell growth, human pancreatic cancer cells (BxPC-3, PANC-1 and AsPC-1) were treated with the varying concentrations (0–1000 nM) of pristimerin for 24 h, 48 h and 72 h, and cell survival was assessed by CCK-8 assay. As shown in Fig. 2, pristimerin treatment resulted in a dose- and time-dependent inhibition of cell growth in all three pancreatic cancer cell lines tested. Inhibitory concentration (IC) 50 values were approximately 652.71 nM, 283.78 nM and 190.54 nM (against BxPC-3) and 969.86 nM, 343.62 nM and 261.05 nM (against PANC-1), and 1261.6 nM, 378.46 nM and 304.57 nM (against AsPC-1) for 24 h, 48 h and 72 h treatment, respectively. These results indicate that pristimerin has potent antiproliferative effects in pancreatic cancer cells.

### Pristimerin Induces G1-phase Cell Cycle Arrest and Alterations in G1-phase Cell Cycle-related Proteins in Pancreatic Cancer Cells

**Figure 3:**
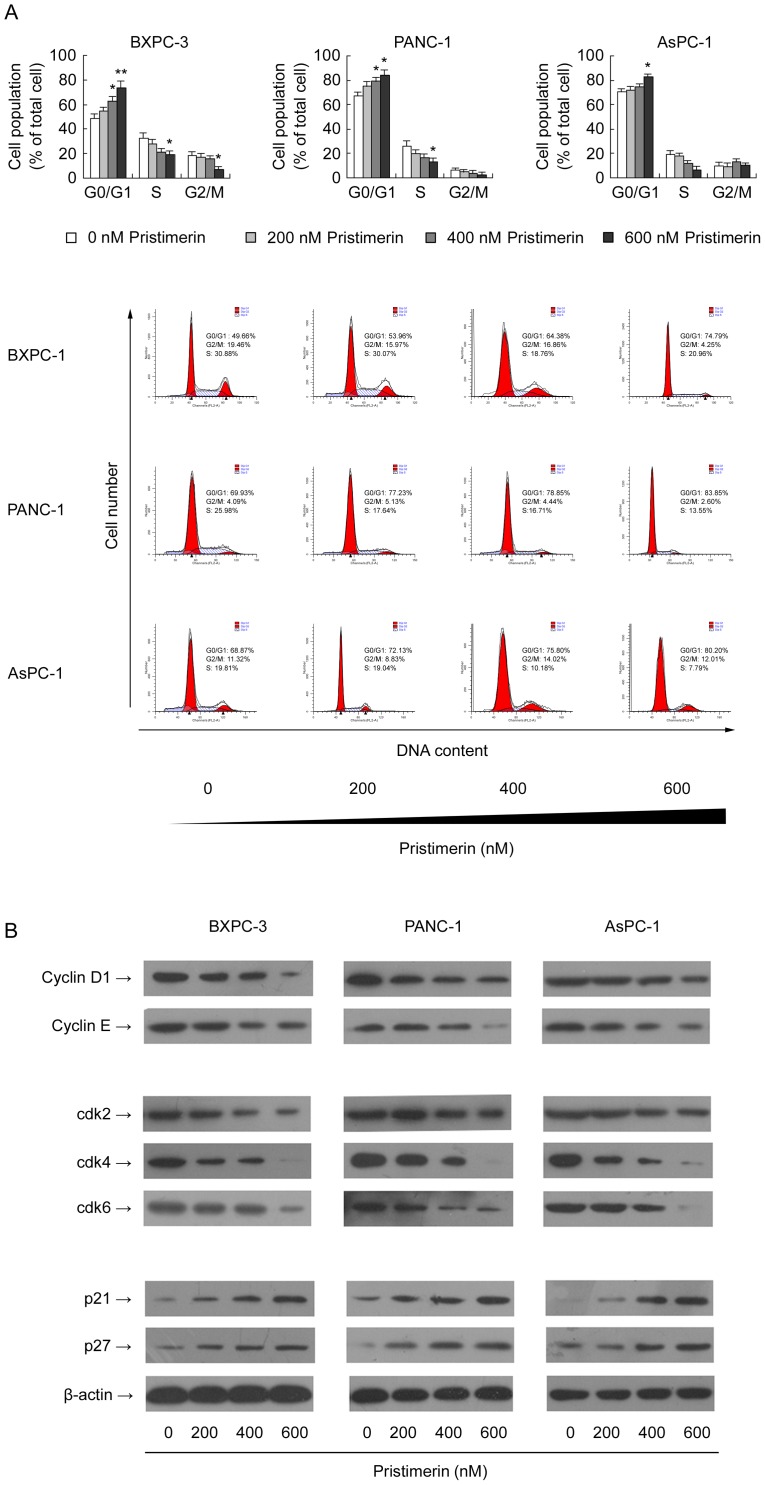
Effect of pristimerin on cell cycle distribution and expression of cell cycle-related proteins. (A) Effect of pristimerin on cell cycle distribution in pancreatic cancer cells. BxPC-3, PANC-1 and AsPC-1 cells were cultured in complete medium and treated with either pristimerin (200, 400 or 600 nM) or DMSO (control) for 48 h. After treatment, cells were collected by trypsinization, washed with ice-cold PBS, and digested with RNase. Cellular DNA was stained with propidium iodide and flow cytometric analysis was performed for the detection of the percentage of cells in the different phases of the cell cycle as described in the Materials and Methods. *P<0.05, compared with control. **P<0.01, compared with control. (B) Effect of pristimerin on the protein level of cyclin D1, cyclin E, cdk 2, cdk 4, cdk 6, WAF1/p21 and KIP1/p27 in pancreatic cancer cells. As detailed in Materials and Methods, pancreatic cancer cells (BxPC-3, PANC-1 and AsPC-1) were treated with either pristimerin (200, 400 or 600 nM) or DMSO (control) for 48 h and then harvested. Total cell lysates were prepared and subjected to SDS-PAGE followed by Western blot analysis for G1 cell cycle regulatory proteins (cyclin D1, cyclin E, cdk2, cdk4, cdk6, WAF1/p21 and KIP1/p27). β-actin was detected as protein loading control. The immunoblots shown here are representative of at least three independent experiments with similar results.

Based on the preliminary assays in which we evaluated the effect of pristimerin on the growth of pancreatic cancer cells, the doses of 200, 400, and 600 nM of pristimerin were selected for further in vitro mechanistic studies. To explore the underlying mechanism of pristimerin-induced growth inhibition of the cells, the effect of pristimerin on cell cycle distribution was studied by Flow cytometric analysis of cellular DNA content. As shown in Fig. 3A, treatment of pancreatic cancer cells with pristimerin for 48 h resulted in a significant dose-dependent arrest of cells in the G1 phase of cell cycle. The G1-phase cell cycle distribution was 48.73, 54.75, 63.29 and 73.62% (in BxPC-3) and 67.23, 75.32, 79.41 and 84.29% (in PANC-1) and 70.79, 72.46, 74.95 and 82.97% (in AsPC-1) at 0, 200, 400 and 600 nM concentrations of pristimerin, respectively. This increase in the contributions of cells in G0–G1 phase was accompanied with a concomitant reduction in the contributions of cells in S phase and G2-M phase of the cell cycle in all three cell lines tested. Taken together, these data suggest that the induction of G1 arrest in pancreatic cancer cells by pristimerin might be responsible for pristimerin-induced cell growth inhibition.

To understand the mechanism underlying G1-phase arrest in pristimerin-treated pancreatic cancer cells, we next investigated the effect of pristimerin on the levels of proteins that regulate the progression of cells in the G1 phase, including WAF1/p21, KIP1/p27, cyclin D1, cyclin E, cdk2, cdk4, and cdk6. As shown in Fig. 3B, treatment of pancreatic cancer cells with pristimerin caused a significant reduction decrease in the protein levels of cyclins D1 and E in a concentration-dependent manner in all three pancreatic cancer cell lines tested. Similarly, a dose-dependent reduction in the expression of cdk2, cdk4, and cdk6 was observed (Fig. 3B). In addition, exposure to pristimerin results in a dose-dependent induction of p21 and p27 in all three pancreatic cancer cell lines tested (Fig. 3B). We suggest that the modulation of cell cycle regulatory proteins by pristimerin may contribute to pristimerin-mediated G1-phase arrest in pancreatic cancer cells.

### Pristimerin Induces Apoptosis in Pancreatic Cancer Cells

**Figure 4:**
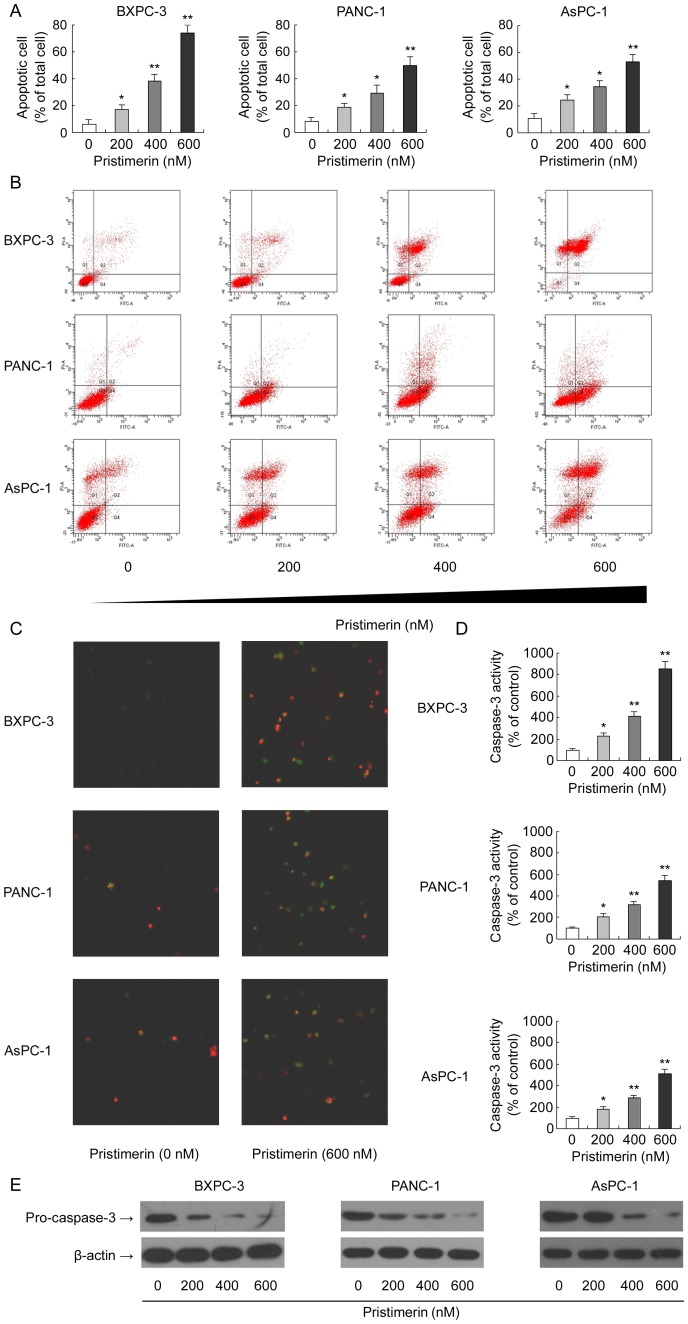
Effect of pristimerin on apoptosis induction in pancreatic cancer cells. (A) Effect of pristimerin on apoptosis induction assessed by Annexin V/PI method using flow cytometry. BxPC-3, PANC-1 and AsPC-1 cells were cultured in complete medium and treated with either pristimerin (200, 400 or 600 nM) or DMSO (control) for 48 h. Apoptosis rate was determined by flow cytometry on annexin V-FITC. (B) Representative dot-plots from cytometrically illustrating apoptotic status in BxPC-3 (upper panel), PANC-1 (middle panel) and AsPC-1 (lower panel) cells. (C) Effect of pristimerin on apoptosis induction assessed by fluorescence microscopy. Cells were also viewed under a fluorescence microscopy. Representative photographs were taken from Annexin V/PI-stained pancreatic cancer cells under certain treatment. (D) Effect of pristimerin on apoptosis induction assessed by caspase-3 activity assay. Cell lysates were assayed for caspase-3 activity as described in “Materials and methods”. (E) Effect of pristimerin on cleavage of caspase-3. As detailed in Materials and Methods, pancreatic cancer cells (BxPC-3, PANC-1 and AsPC-1) were treated with either pristimerin (200, 400 or 600 nM) or DMSO (control) for 48 h and then harvested. Total cell lysates were prepared and subjected to SDS-PAGE followed by Western blot analysis. β-actin was detected as protein loading control. The immunoblots shown here are representative of at least three independent experiments with similar results. *P<0.05, compared with control. **P<0.01, compared with control.

In addition to cell cycle arrest, morphologic observation of pristimerin-treated pancreatic cancer cells indicated that pristimerin-induced growth inhibition could also be associated with the induction of apoptosis. Therefore, we assessed the apoptosis inducing effect of pristimerin in pancreatic cancer cells. Cells were treated with varying concentration of pristimerin (0–600 nM), stained with Annexin V/PI, subjected to flow cytometry to determine the apoptosis rate. As shown in Fig. 4A, treatment of pancreatic cancer cells with pristimerin for 48 h resulted in a significant dose-dependent enhancement in both the early and late stages of apoptosis. The apoptotic indices were 6.3, 17.5, 38.6 and 74.1% (in BxPC-3) and 8.1, 18.7, 29.3 and 49.8% (in PANC-1) and 11.2, 24.5, 34.7 and 52.9% (in AsPC-1) at 0, 200, 400 and 600 nM concentrations of pristimerin, respectively. Laser scanning confocal microscopy confirmed the dose-dependent apoptotic effect as shown in the representative photographs (Fig. 4C). To further explore the effect of pristimerin on cell apoptosis, caspase-3 activities of all three cell lines were also evaluated. pristimerin treatment yielded a dose-dependent enhancement in caspase-3 activity in all three cell lines tested compared with control after 72 h treatment (Fig. 4D). Subsequently, we examined the expression of pro-caspase-3 in all three cell lines tested by western blotting. Pristimerin treatment also down-regulated the expression of pro-caspase-3 in a dose-dependent manner (Fig. 4E). Altogether, our results suggest that pristimerin-induced growth inhibition is in part due to the induction of an apoptosis mechanism.

### Pristimerin Modulates the Levels of Bcl-2 Family Proteins in Pancreatic Cancer Cells

**Figure 5:**
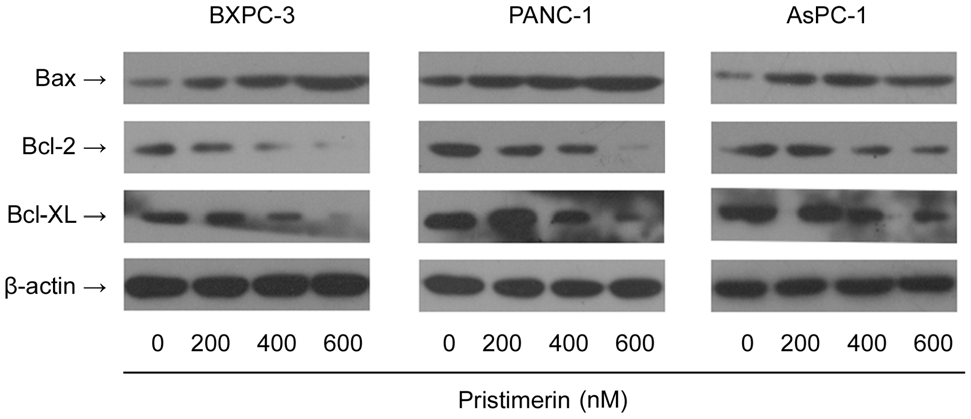
Effect of pristimerin on the protein level of Bcl-xl, Bcl-2 and Bax. As detailed in Materials and Methods, pancreatic cancer cells (BxPC-3, PANC-1 and AsPC-1) were treated with either pristimerin (200, 400 or 600 nM) or DMSO (control) for 48 h and then harvested. Total cell lysates were prepared and subjected to SDS-PAGE followed by Western blot analysis. β-actin was detected as protein loading control. The immunoblots shown here are representative of at least three independent experiments with similar results.

Since Bcl-2 Family proteins play important roles in apoptosis by functioning as promoters (e.g., Bax) or inhibitors (e.g., Bcl-2 or Bcl-xL ) of cell death process, we next studied the changes in the levels of of Bax, Bcl-2 and Bcl-xL in pancreatic cancer cells. Western blot analysis showed that treatment of pancreatic cancer cells for 48 h with increasing concentrations of pristimerin resulted in a dose-dependent decrease in the levels of the anti-apoptotic proteins Bcl-XL and Bcl-2 (Fig. 5). In sharp contrast, the level of pro-apoptotic protein Bax was increased significantly upon treatment with pristimerin in a dose-dependent manner (Fig. 5). Thus, pristimerin treatment can modulate the levels of Bcl-2 family proteins in a manner that would favor increases in the ratios of Bax/Bcl-2 and Bax/Bcl-xL, which may contribute to the observed apoptotic effect of pristimerin.

### Pristimerin Inhibits Translocation and DNA-binding Activity of NF-κB in Pancreatic Cancer Cells

**Figure 6:**
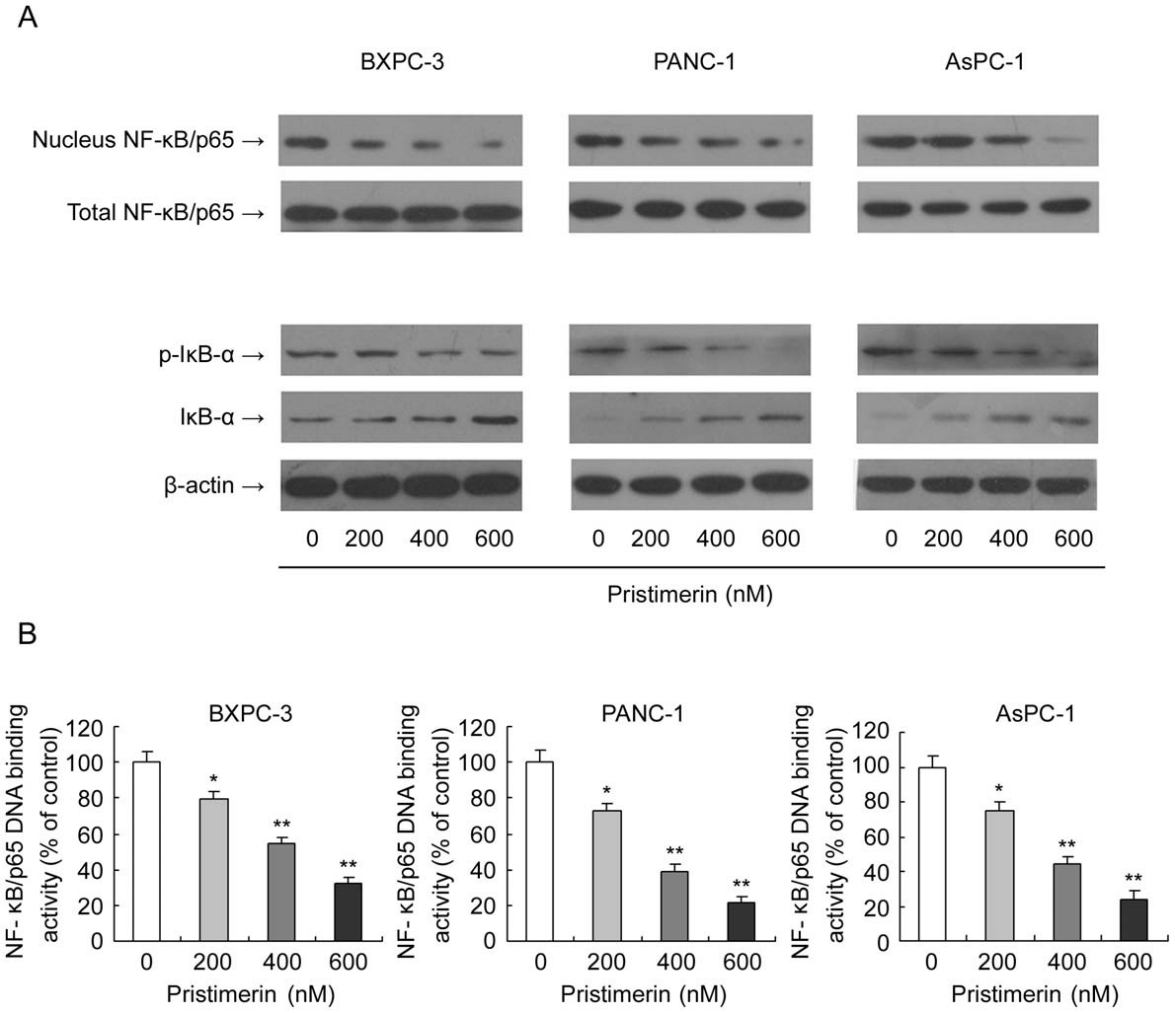
Effect of pristimerin on NF-κB activation in pancreatic cancer cells. (A) Effect of pristimerin on protein levels of NF-κB/p65 in nuclear lysates and total cell lysates of pancreatic cancer cells. As detailed in Materials and Methods, pancreatic cancer cells (BxPC-3, PANC-1 and AsPC-1) were treated with either pristimerin (200, 400 or 600 nM) or DMSO (control) for 48 h and then harvested. Nuclear lysates and total cell lysates were prepared and subjected to SDS-PAGE followed by Western blot analysis for the protein level of NF-κB/p65, p-IκB-α (S32/36) and IκB-α. β-actin was detected as protein loading control. The immunoblots shown here are representative of at least three independent experiments with similar results. (B) Effect of pristimerin on NF-κB/p65 DNA-binding activity in pancreatic cancer cells. As detailed in Materials and Methods, pancreatic cancer cells (BxPC-3, PANC-1 and AsPC-1) were treated with either pristimerin (200, 400 or 600 nM) or DMSO (control) for 48 h and then harvested. Nuclear lysates were prepared and the NF-κB DNA-binding activity was determined using the Trans-Am NF-κB ELISA Kit. *P<0.05, compared with control. **P<0.01, compared with control.

NF-κB has been shown to be constitutively activated in a variety of cancer cells including pancreatic cancer cells and associated with both proliferation and apoptotic resistance. Therefore, We investigated whether pristimerin mediates its effects through modulating NF-κB activity. Employing western blot analysis, we examined the effect of pristimerin on the constitutive expression of NF-κB/p65 and IκB-α in pancreatic cancer cells. Our results demonstrated that pristimerin treatment resulted in a concentration-dependent decrease in NF-κB/p65 protein levels in the nuclear fraction, but there was no change of that in the total protein (Fig. 6A). Consistent with these findings, a decrease in NF-κB protein level was associated with a concentration-dependent decrease in phospho-IκB-α with a concomitant concentration-dependent increase in the cytosolic levels of the IκB-α protein (Fig. 6A). To further support this observation, we examined the effect of pristimerin on the DNA-binding activity of NF-κB/p65 using a specific NF-κB/p65 ELISA and found pristimerin treatment resulted in a significant dose-dependent reduction in DNA-binding activity of NF-κB/p65 in all three pancreatic cancer cells (Fig. 6B). Taken together, our results suggest that treatment with pristimerin results in a significant suppression of constitutive NF-κB activation in pancreatic cancer cells.

### Pristimerin Potentiates the Cytotoxic Effect of Gemcitabine by Reducing Cell Viability and Promoting Apoptosis

Gemcitabine, the best chemotherapeutic agent available for the treatment of advanced pancreatic cancer, alone is not very effective and is associated with systemic toxicity and chemoresistance. Because the activation of NF-κB has been shown to promote gemcitabine resistance in pancreatic cancer, we assessed whether pristimerin can potentiate the effect of gemcitabine in these cell lines.

**Figure 7:**
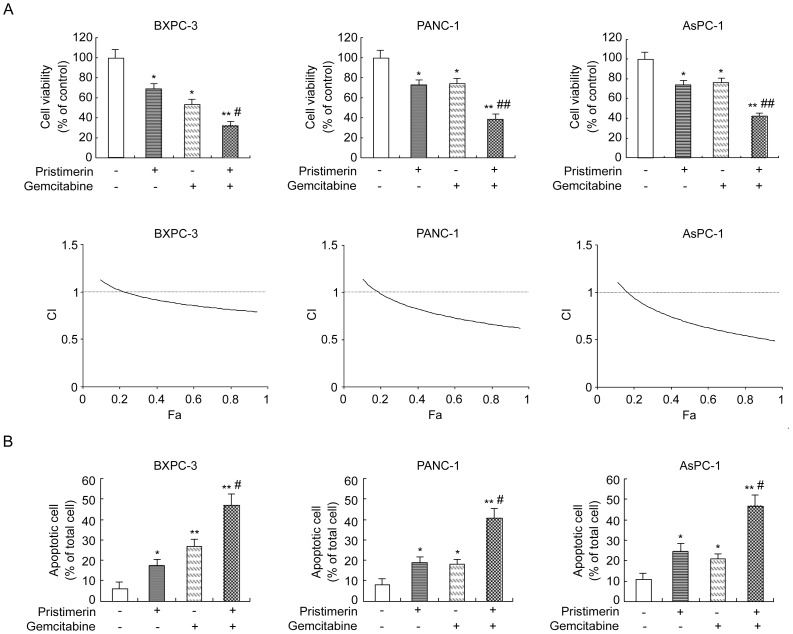
Potentiation of the effect of gemcitabine by pristimerin in pancreatic cancer cells. (A) Potentiation of gemcitabine-induced cell growth inhibition by pristimerin. Cells were grown in the absence or presence of pristimerin (200 nM), gemcitabine (500 nM) or their combination for 48 h. The viability of cells was measured by CCK-8 assay as described in Materials and Methods. Combination index (CI) versus fraction affected (Fa) plots obtained from median-effect analysis of Chou-Talalay. CI values: >1, antagonism; 1, additivity; <1, synergism. (B) Potentiation of gemcitabine-induced apoptosis by pristimerin. Cells were grown in the absence or presence of pristimerin (200 nM), gemcitabine (500 nM) or their combination for 48 h. Apoptosis rate was determined by flow cytometry on annexin V-FITC. *P<0.05, compared with control. **P<0.01, compared with control. ^#^P<0.05, compared with single gemcitabine group.^ ##^P<0.01, compared with single gemcitabine group.

CCK-8 assay showed that treatment with either pristimerin (200 nM) or gemcitabine (500 nM) alone for 48 hours resulted in only 31.5% or 46.7% loss of viability of BXPC-3, and 27.2% or 25.9% loss of viability of PANC-1, and 26.3% or 23.6% loss of viability of AsPC-1, respectively. However, the combination of pristimerin and gemcitabine resulted in the loss of 68.1%, 61.7% or 57.8% of viable cells in either cell type investigated, respectively (Fig. 7A). The nature of the interaction between pristimerin and gemcitabine was quantitated by using the Chou/Talalay median-effect equation method to derive a combination index. A CI <1 denotes a synergistic effect, and the CI range values for the combination of pristimerin and gemcitabine in all three human pancreatic cancer cell lines were 0.5 to 0.9 for fractional effect corresponding to 0.3 to 0.9 (Figure 7A), indicating that the combination of pristimerin and gemcitabine have synergistic effects on inhibiting cell growth in all cell lines tested.

Annexin V assay showed that single gemcitabine (500 nM) increased the rate of apoptosis from 6.3% to 26.9% in BxPC-3 cells and from 8.1% to 17.9% in PANC-1 cells and from 11.2% to 20.8% in AsPC-1 cells, while single pristimerin (200 nM) increased the rate of apoptosis from from 6.3% to 17.5% in BxPC-3 cells and from 8.1% to 18.7% in PANC-1 cells and from 11.2% to 24.5% in AsPC-1 cells. Furthermore, gemcitabine in combination with pristimerin led to an increased apoptosis compared with single-agent treatment in respective cells (46.8%, 40.5% and 46.7%, respectively) (Fig. 7B). These results together indicate that pristimerin augments the cytotoxic effect of gemcitabine in pancreatic cancer cells.

**Figure 8:**
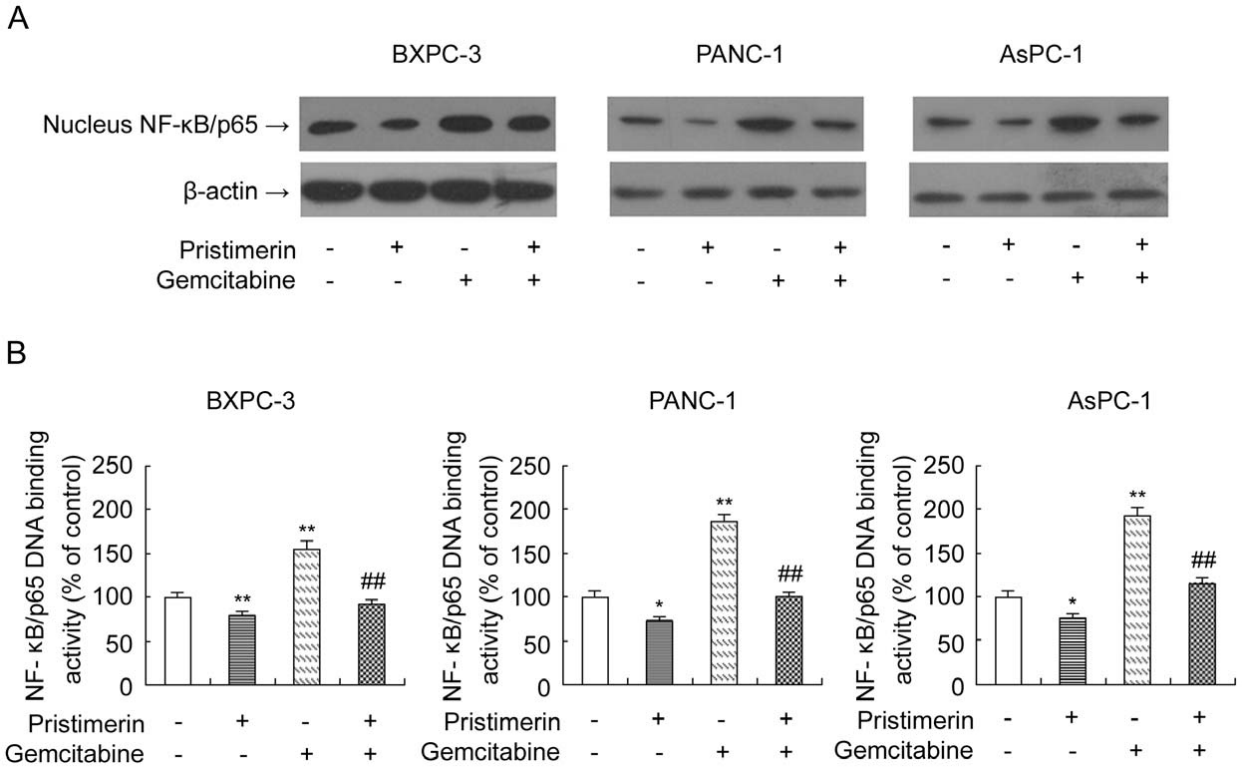
Abrogation of constitutive and gemcitabine-induced NF-κB activation by pristimerin in pancreatic cancer cells. (A) Effect of pristimerin and gemcitabine on protein levels of NF-κB/p65 in nuclear lysates and total cell lysates of pancreatic cancer cells. As detailed in Materials and Methods, pancreatic cancer cells (BxPC-3, PANC-1 and AsPC-1) were grown in the absence or presence of pristimerin (200 nM), gemcitabine (500 nM) or their combination for 48 h and then harvested. Nuclear lysates and total cell lysates were prepared and subjected to SDS-PAGE followed by Western blot analysis for the protein level of NF-κB/p65. β-actin was detected as protein loading control. The immunoblots shown here are representative of at least three independent experiments with similar results. (B) Effect of pristimerin and gemcitabine on NF-κB/p65 DNA-binding activity in pancreatic cancer cells. As detailed in Materials and Methods, pancreatic cancer cells (BxPC-3, PANC-1 and AsPC-1) were grown in the absence or presence of pristimerin (200 nM), gemcitabine (500 nM) or their combination for 48 h and then harvested. Nuclear lysates were prepared and the NF-κB DNA-binding activity was determined using the Trans-Am NF-κB ELISA Kit. *P<0.05, compared with control. **P<0.01, compared with control. ^#^P<0.05, compared with single gemcitabine group. ^##^P<0.01, compared with single gemcitabine group.

Next, We assessed the effect of pristimerin and gemcitabine on NF-κB activity to determine whether this benificial effect of pristimerin and gemcitabine is associated with the inhibition of NF-κB activation. Our data showed that BxPC-3, PANC-1 and AsPC-1 cells expressed constitutively active NF-κB DNA-binding activity and gemcitabine alone further induced NF-κB DNA-binding activity compared to control (Fig. 8A and B). Interestingly, in all three cells, pristimerin was able to reduce NF-κB DNA-binding activity no matter with, or without gemcitabine (Fig. 8A and B). These results indicate that pristimerin not only reduces constitutively active NF-κB DNA-binding activity in unstimulated conditions but also inhibits gemcitabine-induced NF-κB activation, which could be responsible for the potentiating effect of pristimerin on gemcitabine against pancreatic cancer cells.

## Discussion

The evaluation of naturally occurring dietary compounds may indicate novel approaches for the treatment of pancreatic cancer, which remains one of the most lethal cancers despite tremendous scientific efforts [2]. In our present work, we found that pristimerin exerted significant growth inhibitory effects on pancreatic cancer cells (BxPC-3, PANC-1 and AsPC-1) via induction of G1 arrest and apoptotic cell death. In addition, pristimerin enhanced chemosensitivity to gemcitabine in pancreatic cancer cells. Our data indicated that the anti-proliferative and chemosensitization effect of pristimerin on pancreatic cancer cells may be mediated through the inhibition of NF-κB activity and alteration of cell cycle- and apoptosis-related proteins.

Modulation of cell cycle progression in cancer cells is regarded as an appreciated target for the treatment of human malignancies since various studies have shown that aberration of cell cycle regulators is a common feature of human cancer [23], [24], [25]. Our in vitro results demonstrated that treatment of pancreatic cancer cells with pristimerin resulted in dose-dependent arrest of cells in G1 phase. The passage through the cell cycle, in eukaryotes is governed by complexes containing cyclins, the regulatory units, with cyclin-dependent kinases (Cdks), the catalytic units [26]. Cyclins D and E along with cdk2, cdk4, or cdk6 play important roles in the progression of cells through the G1 phase of the cell cycle [27]. The deregulation of G1 phase cell cycle regulators is believed to promote the aberrant proliferation of cancer cells. Overexpression of cyclins and cdks can provide cancer cells with a selective growth advantage [28]. Therefore, targeting cyclin/cdk complexes is considered to be a promising effective strategy for the treatment of cancer. During the progression of the cell cycle, the cdk-cyclin complexes are inhibited via binding to Cdks inhibitors (ckis) such as CIP/KIP and INK4 families of proteins [29]. WAF1/p21 and KIP1/p27, proteins of Cip/Kip family, block cell cycle progression by inhibiting the activity of Cyclin E-Cdk2 complexes that normally promote G1-S phase progression. The observed inhibitory effects of pristimerin on cyclin D1, cyclin E, cdk2, cdk4 and cdk6, along with its upregulating effects on WAF1/p21 and KIP1/p27 in pancreatic cancer cells suggest its potential in disruption of the uncontrolled cell cycle progression. Altogether, these data indicate that pristimerin arrests pancreatic cancer cells in G1 phase of cell cycle via modulating cell cycle regulators suggesting one of the mechanisms by which pristimerin may act to suppress cancer cell growth and induce apoptotic cell death.

G1-phase cell cycle arrest creates an opportunity for cells to either undergo repair or enter the apoptotic pathway to maintain tissue homeostasis and eliminate the mutated neoplastic and hyperproliferating neoplastic cells from the system. In recent years, the concept of cell cycle regulation-mediated apoptosis has gained increasing attention, and has emerged as an ideal way of cell growth inhibition [30], [31]. In this aspect, pristimerin seems to be a potent chemotherapeutic agent as it could selectively/preferentially eliminate cancer cells by causing cell cycle arrest and/or inducing apoptosis [20]. In the present study, flow cytometry data showed that treatment of pancreatic cancer cells with pristimerin resulted in signifiant induction of apoptosis, which was further verified by cleavage of caspase-3, fluorescence microscopy and caspase-3 activity. Collectively, these results clearly demonstrate that the cytotoxic effect of pristimerin in pancreatic cancer cells is mediated via induction of cell cycle arrest and apoptosis.

Members of the Bcl-2 family of proteins have a central role in controlling the apoptotic pathway. Some proteins within this family, including Bcl-2 and Bcl-XL, suppress apoptosis, while others such as Bax and Bak promote apoptosis [32], [33], [34] The pro-apoptotic proteins and anti-apoptotic proteins of the Bcl-2 family could form heterodimers, resulting in mutual neutralization of the bound pro-and anti-apoptotic effects. Hence, alterations in the levels of anti- and pro-apoptotic Bcl-2 family proteins are critical for the induction of apoptosis. We found that treatment of pancreatic cancer cells with pristimerin resulted in an increase in the expression of Bax protein and a decrease in the expression of Bcl-2 and Bcl-xL thereby increasing the ratios of Bax/Bcl-2 and Bax/Bcl-xL in favor of apoptosis. Therefore, it seems logical to postulate that upregulation of Bax and downmodulation of Bcl-2 and Bcl-XL may be responsible for the observed apoptotic effect of pristimerin.

Certain naturally occurring bioactive compounds with chemopreventive properties in pancreatic cancer cells such as benzyl isothiocyanate, honokiol, curcumin, dihydroartemisinin and fisetin have been shown to inhibit cell proliferation and induce apoptosis through a NF-κB - dependent mechanism [35], [36], [37], [38], [39]. Nuclear factor-κB (NF-κB),which plays a critical regulatory role in the expression of genes involved in inflammation, cell proliferation, invasion, angiogenesis, metastasis, suppression of apoptosis, is constitutively activated in a variety of cancer cells including pancreatic cancer cells [6], [7], [8]. NF-κB is a homo- or heterodimer composed of members of the NF-κB family, including NF-κB1 (p50), NF-κB2 (p52), RelA (p65), RelB and c-Rel [40]. The most common form is a dimmer of RelA (p65) and NF-κB1 (p50), and is sequestered in the cytoplasm in an inactive state through interaction with an endogenous inhibitor IκB [41]. Following cellular stimulation, IκB undergoes phosphorylation- and ubiquitination-dependent degradation, thereby allowing active NF-κB to translocate into the nucleus where it binds with specific response elements in the DNA sequences to switch on gene transcription including antiapoptotic (Survivin, Bcl-xL, Bcl-2), proliferative (Cyclin D1), proinflammatory (COX-2), invasion [matrix metalloproteinase 9 (MMP-9)], and angiogenic [vascular endothelial growth factor (VEGF)] genes [7], [12], [14], [42], [43]. It is becoming increasingly clear that agents that could suppress NF-κB activation have potential for the treatment of cancers including pancreatic cancer. Data presented herein indicated that pristimerin treatment resulted in a dose-dependent inhibition of NF-κB activity with subsequent increase in the protein expression of IκB-α. We also found that pristimerin decreased the expression of NF-κB-regulated gene product Bcl-2, Bcl-XL and cyclin D1 in pancreatic cancer cells, thus suppressed the growth of pancreatic cancer cells. Taken together, these data suggest that pristimerin-mediated down-modulation of NF-kB activity may be an important mechanism for the growth inhibitory effects of pristimerin on pancreatic cancer cells.

Because NF-κB activation has been also associated with chemoresistance [15], [44]. we next examined the effect of pristimerin on sensitivity to gemcitabine, the best chemotherapeutic agent available for the treatment for advanced pancreatic cancer. Our data demonstrated that pristimerin could potentiate the effect of gemcitabine against human pancreatic cancer cells by reducing cell viability and promoting apoptosis. In addition, our data demonstrated that gemcitabine alone obviously enhanced NF-κB DNA-binding activity and led to a increased rate of proliferation and a decreased rate of apoptosis, indicating that the potentiating effect of pristimerin on gemcitabine may result from inhibition of NF-κB, as also demonstrated by our previous studies and others that the inhibition of NF-κB could augment the cytotoxic effect of multiple chemotherapeutic agents [1], [15], [22].

**Figure 9:**
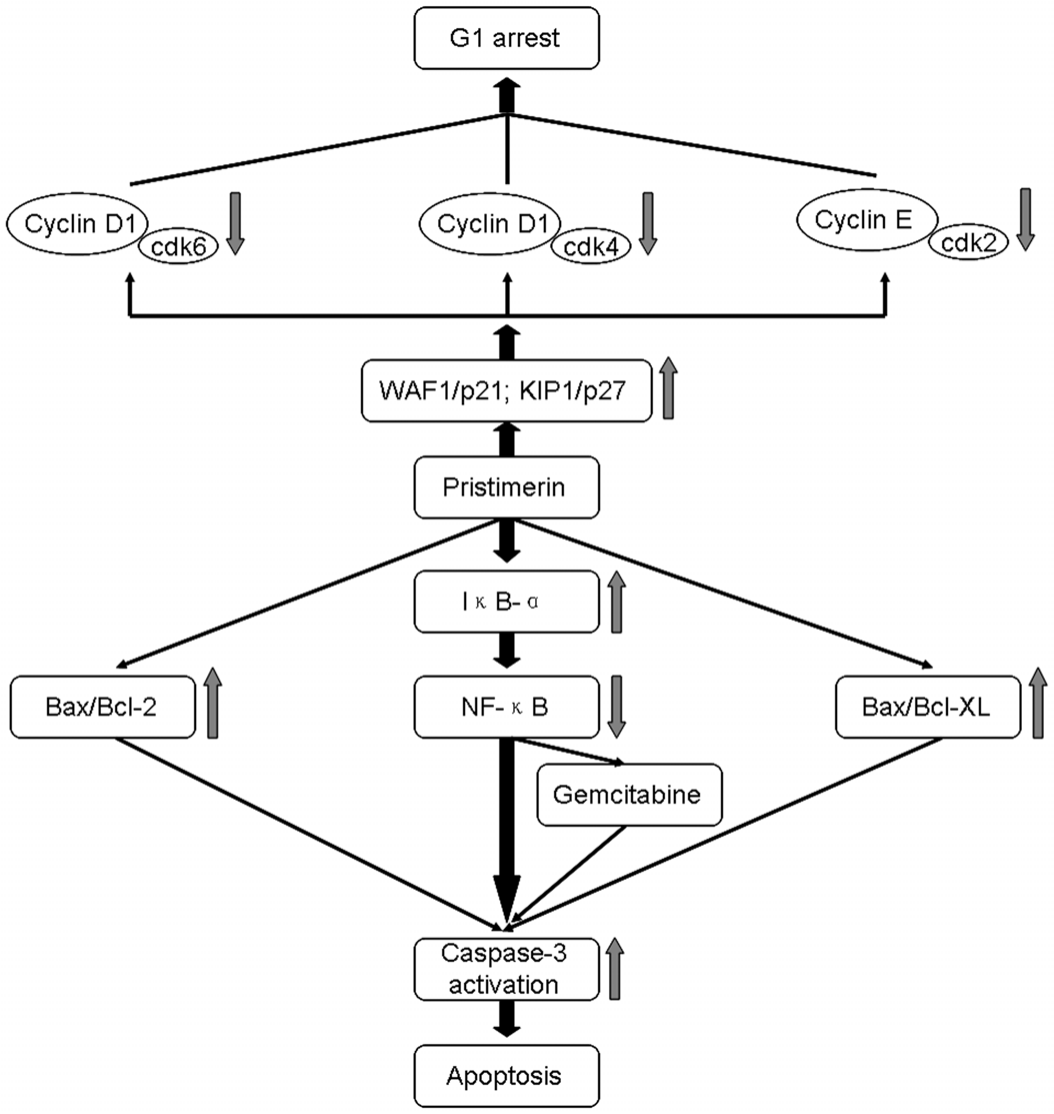
Proposed mechanisms of pristimerin-mediated G1 arrest, apoptosis and chemosensitivity of human pancreatic cancer cells.

Overall, the present study demonstrated that pristimerin inhibits proliferation, induces G1–phase arrest and apoptosis, and potentiates the cytotoxic effect of gemcitabine in pancreatic cancer cells. In addition, our results provide mechanistic evidence that the antiproliferative, proapoptotic and chemosensitive effects of pristimerin may be associated with inhibition of NF-κB pathway. Based on the outcome of the present study, the mechanisms by which pristimerin causes G1 arrest, induces apoptosis, and enhances the chemosensitivity to gemcitabine in pancreatic cancer cells are summarized in Fig 9. Taken together, we suggest pristimerin have potential as a chemotherapeutic drug or as a chemosensitizer to improve the therapeutic index of gemcitabine in the treatment of pancreatic cancer.

## Materials and Methods

### Materials

Pristimerin were purchased from Sigma-Aldrich, Inc. (St. Louis, MO, USA). Gemcitabine (Gemzar, France) was purchased from Lily France, Fegersheim, France. The primary antibodies used in this experiment, unless otherwise stated, were obtained from Santa Cruz Biotechnology (Carlsbad, CA, USA). Nuclear Extract Kit and Trans-AM NF-κB p65 ELISA Kit were obtained from Active Motif (Carlsbad, CA, USA).

### Cell Culture

The human pancreatic cancer cell lines PANC-1, BxPC-3 and AsPC-1 were obtained from the American Type Culture Collection (Rockville, USA) and were cultured in DMEM and RPMI 1640 medium, respectively, supplemented with fetal bovine serum (10%), penicillin (100 U/ml) and streptomycin (100 µg/ml) (Irvine Scientific, Irvine, CA). All cells were maintained at 37°C in humidified air with 5% CO_2._ (all reagents were from HyClone China Ltd., China). Mycoplasma contamination was tested using the Mycoplasma Stain Assay Kit (Beyotime Institute of Biotechnology, Beijing, China). None of the cell cultures were contaminated with mycoplasma.

### Treatment of Cells

Pristimerin dissolved in DMSO [final concentration, 0.1% (v/v)] was used for the treatment of cells. The subconfluent cells (60–70% confluent) were treated with various concentrations of pristimerin alone, gemcitabine alone or their combinations (as indicated in the figure legends) in complete cell culture medium and cells treated with 0.1% DMSO served as control. The following experiments were repeated thrice.

### CCK-8 Assay

Cell viability in the treated cells was determined by using Cell Counting Kit-8 (CCK-8) kit (Dojindo Laboratories, Kumamoto, Japan) following the instructions outlined by the manufacturer and as previously described by us [22]. The assay uses a tetrazolium salt, WST-8, which can be converted into the water-soluble formazan dye on bioreduction by cellular dehydrogenases. Briefly, cells were plated at a density of 3–5×10^3^ cells/well with 200 µl of medium in 96-well microtiter plates. After treatment, CCK-8 solution (10 µl) was added to each well and the plates were incubated at 37°C for 90 min. The absorbance of the cell suspension was measured with a microplate reader at a wavelength of 450 nm. The highest concentration of pristimerin and gemcitabine as single agents and in combination do not interfere with the CCK-8 assay reagents, in the absence of cells (data not shown). Medium containing 10% CCK-8 served as a control.

### Combination Index Analysis

For evaluating the combination effect of pristimerin and gemcitabine, the combination index (CI) isobologram method of Chou and Talalay was used [21]. This commonly used analysis involves plotting concentration-effect curves for each agent and for multiply diluted fixed-ratio combinations by using the median–effect equation and the combination index equation. A value of CI less than, equal to or greater than 1 indicates synergism, additivity and antagonism, respectively. The combination index values were calculated at multiple effect levels and the isobolograms plotted.

### DNA Cell Cycle Analysis

The methodology has been described previously [22]. Subconfluent cells were treated with pristimerin (0–600 nM, 48 h) in complete medium. The cells were then collected by trypsinization, washed twice with ice-cold PBS, and fixed for at least 4 h by addition of 2 mL of ice-cold 70% ethanol/30% PBS at 4°C. The ethanol was subsequently removed after centrifugation and about 1×10^6^ cells were re-suspended in 800 µl of PBS, 100 µl of ribonuclease A (500 µg/ml PBS) and 100 µl of propidium iodide (500 µg/ml PBS) at room temperature in the dark for 30 min. Flow cytometric analysis was performed using FACScan (Becton Dickinson) for the detection of the percentage of cells in various phases of the cell cycle.

### Cell Apoptosis Assay

The percentage of cells actively undergoing apoptosis was determined by flow cytometry using an Annexin V assay kit following the instructions outlined by the manufacturer and as previously described by us [22]. Briefly, after treatment, cells were harvested with trypsin, washed in PBS, and counted. 1×10^5^ cells were then resuspended in binding buffer at a concentration of 1 × 10^6^ cells/ml. Next, Ten µl of Annexin V and 5 µl of PI were added, and the cells were incubated at room temperature for at least 15 minutes in the dark. After incubation, the percentage of apoptotic cells was analyzed by flow cytometry (Epics Altra II, Beckman Coulter, USA). Cells were also visualized under a laser scanning confocal microscope (LSM-510, Carl Zeiss Jena GmbH, Jena, Germany) to detect apoptosis.

### Caspase-3 Activity Assay

The activity of caspase-3 was measured using a Caspase-3 Activity Kit (Beyotime Institute of Biotechnology, Haimen, Jiangsu, China) following the instructions outlined by the manufacturer and as previously described by us [22]. Briefly, cells were harvested after being treated as described above, washed with ice-cold PBS, resuspended in lysis buffer (100 µl per 2×10^6^ cells), left on ice for 15 min and then centrifuged at 18,000 g at 4°C for 10 min. Assays were performed on 96-well microtitre plates by incubating the mixture composed of 10 µl protein of cell lysate, 80 µl reaction buffer and 10 µl caspase-3 substrate (Ac-DEVD-pNA) (2 mM) at 37°C for 4 h. The activity of caspase-3 in the samples was quantified by using a Microplate Reader at an absorbance of 405 nm. The activity of caspase-3 was expressed as percentage of enzyme activity compared to control.

### Western Blotting

Protein extracts and Western blotting were done as described previously [22]. Briefly, cells were washed twice in PBS, sonicated in RIPA buffer (Beyotime Institute of Biotechnology, Beijing, China) and homogenized. Debris was removed by centrifugation at 12,000 g at 4°C for 10 min and protein concentration was determined using the BCA Protein assay according to the manufacturer’s instructions. Samples containing equal amounts of protein (50 µg) were separated by electrophoresis on 10% or 15% polyacrylamide SDS gels (100 V for 1 to 2 hours) and transferred to polyvinylidene difluoride (PVDF) membranes by electroblotting (100 V for 1 hour at 4°C). The running time and voltage as well as transfer time and voltage may require some optimization depending on the circumstances. The membranes were then blocked by incubating with 5% skim milk in TBST buffer (TBS plus 0.1% Tween 20) for 2 hour and incubated with the appropriate primary antibody with gentle agitation overnight at 4°C. The membranes were then washed several times and incubated with the appropriate horseradish peroxidase–conjugated secondary antibody (Santa Cruz Biotech, Santa Cruz, CA, USA) for 1 hour at room temperature. The membranes were incubated with appropriate secondary antibody conjugated with horseradish peroxidase (Santa Cruz Biotech, Santa Cruz, CA, USA) for 1 hour at room temperature. The membranes were then washed and protein bands were visualized the enhanced chemiluminescence (ECL) kit followed by exposure of the membrane to X-ray film. β-actin was simultaneously determined as a loading control.

### NF-κB DNA-binding Activity Assay

The NF-κB DNA-binding activity was determined using the Trans-Am NF-κB/p65 ELISA Kit following the instructions outlined by the manufacturer. Nuclear and cytosolic fractions were prepared from cells by using Nuclear Extraction Kit following the instructions outlined by the manufacturer. Briefly, nuclear lysate protein from each group was added to a 96-well plate with an immobilized oligonucleotide containing the specific consensus sequence (5′ -GGGACTTTCC-3′) for NF-κB/p65 binding. Following incubation for 1 h at room temperature to facilitate the binding, a primary antibody specific for NF-κB was added to each well, followed by a horseradish peroxidase-conjugated secondary antibody. The absorbance was taken at 450 nm using a ELISA plate reader.

### Statistical Analysis

The data obtained were expressed as the mean value ± standard deviation (SD), and a student’s t test was used to assess statistical significance. A value of less than 0.05 (P<0.05) was considered significant.
